# Techniques to improve the maintenance of a laboratory colony of *Nyssomyia neivai* (Diptera: Psychodidae)

**DOI:** 10.1186/s13071-015-1035-7

**Published:** 2015-08-16

**Authors:** Thais Marchi Goulart, Camila Feitosa de Castro, Vicente Estevam Machado, Flávia Benini da Rocha Silva, Mara Cristina Pinto

**Affiliations:** Departamento de Zoologia Animal, Universidade Estadual de Campinas, Campinas, SP 13083-970 Brazil; Departamento de Ciências Biológicas, Faculdade de Ciências Farmacêuticas, UNESP - Univ Estadual Paulista, Araraquara, SP 14801-902 Brazil

**Keywords:** Sand flies, Phlebotomine, Immature stages, Larval diet, *Nyssomyia neivai*, Substrates, Sterilized diet

## Abstract

**Background:**

The most critical phase in sand fly colonization is the high mortality in the larval instars. In this study, we sought out strategies for improving the colonization of *Nyssomyia neivai*, one of the vectors of cutaneous leishmaniasis agent in South America.

**Methods:**

A colony of *Ny. neivai* was established in the laboratory from a field population, and the productivity of adults was evaluated considering carrying capacity, diet for larvae and surface for oviposition.

**Results:**

The highest emergency rate of adults was achieved with the fewest couples inside 150 mL rearing chambers on a sterilized diet made of rabbit feces, rabbit food, soil and fish food and with vermiculite as a substrate for oviposition and the development of larvae.

**Conclusion:**

Our data on *Ny. neivai* colonization showed that the best adult productivities were achieved with fewer couples inside the rearing chambers; smaller rearing containers of 150 mL (due to less fungi growth); sterilized diet made of rabbit feces, rabbit food, soil and fish food; and vermiculite as the substrate for oviposition and development of larvae.

## Background

Colonies of insect vectors are suitable for a wide range of investigations, from basic biological aspects to applied research. Despite the importance of laboratory colonies of the vectors of leishmaniasis agent, the difficulty in providing the specific requirements of the more than 900 sand fly species [1] explain why less than 60 species have been successfully reared in the laboratory [2]. The main problem in rearing sand flies is the high mortality during the immature phases. Sand fly larvae are detritivores, and the conventional diet for larvae in laboratory colonies consists of a variety of substrates that are mainly rich in fungal, bacterial and plant material [3]. Although some studies have focused on diets for sand fly larvae, there is not a universal recipe for all species. There have been discussions on whether the different alimentary requirements of phlebotomine larva can contribute to reducing the competition among sand fly species under natural conditions [4].

*Nyssomyia neivai* is incriminated as one of the vector of cutaneous leishmaniasis agents in South America [5–8].

The aim of this research was to evaluate the carrying capacity, attraction of the immature stages of *Ny. neivai* to different diets, survival of the larvae on distinct diets and different rearing surfaces.

## Methods

### Collection and maintenance of sand flies

Sand flies were collected in Santa Eudóxia, São Paulo state - Brazil (along the edges of the Mogi Guaçu River) on the wall of a house, using a manual aspirator between 18:00 and 23:00 h. In the laboratory, the sand flies were maintained in cages covered with voile (30x30x30 cm) at 26 ± 1 °C, 80–90 % humidity, and a 12:12 (L:D) photoperiod. The sand flies had access to a piece of cotton soaked in a 30 % sucrose solution. The non-bloodfed field-collected females were exposed to BALB/c mice as a blood source. The bloodfed females were individually transferred to vials to oviposition. After oviposition, sand flies were clarified and identified under an optical microscope following the identification key by Galati [9]. *Ny. neivai* eggs were placed in a 150 mL polystyrene container filled with 2 cm of plaster of Paris on its bottom (oviposition container). The following experiments were carried out in oviposition containers that were kept in the dark at 26 ± 1 °C, 80–90 % humidity.

### *Ny. neivai* carrying capacity in rearing containers of different sizes

Groups of bloodfed females and an equal number of males were selected and transferred to 250 mL and 150 mL polystyrene oviposition chambers. Pairs of male and female sand flies were placed in 250 mL containers in groups of 5, 10, 15, 20, 25 and 30. For the 150 mL containers, 5, 10 and 15 bloodfed females were placed to verify the carrying capacity. The experiments were repeated four times. The chambers were observed daily to check the humidity levels, the mortality of gravid females and the number of eggs. The sucrose solution was changed daily. At the end of the experiments, the productivity of adults was evaluated for each group.

### Attractiveness of different diets for *Ny. neivai* larvae

In the laboratory, we use diets made of rabbit feces, rabbit food from Evialis®, fish food from Alcon® Colours and soil (modified from a personal communication with José Carlos Miranda). We tried other combinations to evaluate their attractiveness for larvae.

Fifty eggs of *Ny. neivai* were placed in the center of a 250 mL plastic container. The lining of plaster of Paris in the container bottom was divided into four quadrants, and over each of them a specific diet was added on 7^th^ day after oviposition: diet 1 (traditional diet - used in routine rearing of sand flies), diet 2 (rabbit feces and soil), diet 3 (rabbit feces, soil and fish food) and diet 4 (soil and mud – Barretto, 1942). The number of larvae on each quadrant was observed and counted daily until the pupal phase. The experiments were repeated four times.

Diet preparation: rabbit feces were collected from the Veterinary Institute of Universidade de São Paulo (USP) and dried by solar heat for 3 days. The feces, rabbit food, fish food and soil were triturated and sieved through 0.297 mm, 0.125 mm and 0.074 mm meshes. All of the components, except the fish food, were sterilized in an autoclave.

### Attractiveness and development of *Ny. neivai* larvae on two different diets

One hundred eggs were placed in the center of each container (250 mL). The plaster of Paris was divided into four quadrants, and over each one, of the two different diets was added: diet 1 (rabbit feces, rabbit food, fish food and soil) and diet 2 (multimixture flour - a food supplement for pregnant women and children to combat malnutrition - containing rice and wheat bran, wheat flour, corn meal, sesame, soybean, linseed, *Moringa oleifera* leaves, sunflower or pumpkin seeds and cassava leaf powder). Neighbouring quadrants received different diets. The number of larvae on each quadrant was observed and counted daily until the pupal phase.

To analyse which diet was better for the development of the insects, each diet was added in different containers with 100 eggs each. The diet was added according to the needs of the larvae in different instars. Both experiments were repeated four times.

### Development of immature stages on non-sterilized and sterilized diets

One hundred eggs were placed in the center of clean containers (150 mL) and Diet 1 (rabbit feces, rabbit food, fish food and soil) was added daily in the following way: non-sterilized and sterilized (by autoclave or tyndallization). The tests were carried out until the adult phase and were replicated five times.

### Evaluation of different substrates for rearing the colony

The substrates that were evaluated individually were plaster of Paris and plaster with a fine layer of vermiculite over it. Five engorged females and five males were placed in the oviposition containers (150 mL). A piece of plaster of Paris was put in the center of the container and used as resting spot. A piece of cotton soaked in a 30 % sucrose solution was offered to the couples. Emerged adults were counted, and the results were analysed. The experiments were repeated 10 times.

### Statistical analysis

Data were analysed by Analysis of Variance (ANOVA) and Tukey’s tests or t-tests performed using GraphPad Prism version 5.00.

### Ethical approval

This study was carried out in accordance with the Ethical Principles of Animal Experimentation, approved by the Committee on the Ethics of Animal Experiments at State University of Campinas (Protocol number 2951–1).

## Results

### *Ny. neivai* carrying capacity in rearing containers of different sizes

The female mortality before oviposition was random and was not dependent on the number of couples in the containers (data not shown). The mean number of eggs per female varied between the different sizes of the rearing containers. For the smallest containers (150 mL), five couples presented the highest mean number of eggs per female (67.2 eggs; *p* < 0.05). For the largest containers (250 mL), there was no statistical difference among the number of couples in the pots (from 47.1 to 56.5 eggs; *p* > 0.05) (Table 1).

**Table 1:** Mean number of eggs per females and standard error in rearing chambers of 150 mL (5, 10 and 15 couples) and 250 mL (5, 10, 15, 20, 25, 30 couples)

Containers	Number of couples per chamber					
	5	10	15	20	25	30
150 mL	67.2a	44.4b	41.6b	-	-	-
	(±5.2)	(±4.4)	(±4.6)			
250 mL	47.1a	50.5a	51a	51.1a	56.5a	51a
	(±10.6)	(±3.3)	(±2.0)	(±6.4)	(±3.9)	(±5.5)

Means with different letters on the same line are significantly different (P<0.05)

**Fig. 1:**
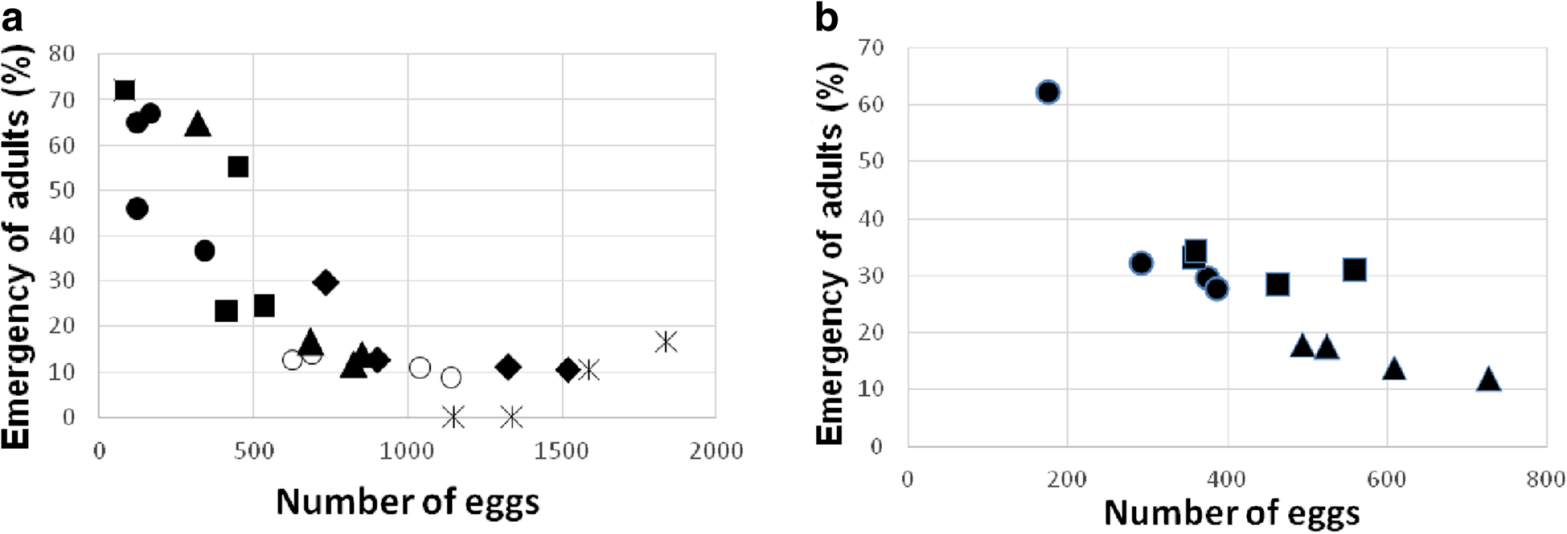
Percentages of adult emerged per number of eggs in different size containers. **a** ● five couples, ^▄^ ten couples, ▲fifteen couples, ○ twenty couples, ♦ twenty five couples, _*_ thirty couples in 250 mL and **b** ● five couples, ^▄^ ten couples, ▲fifteen couples in 150 mL containers

The number of adults emerged was negatively correlated with the number of eggs per rearing container. Adult emergency rates were over 50 % in the 250 mL containers that contained approximately 400 eggs. For 150 mL containers, the emergency rates were 30 % (around 600 eggs per pot). The best results for both the smaller and larger containers were seen with five to ten insect couples (Fig. 1).

### Attractiveness of different diets to *Ny. neivai* larvae

**Fig. 2:**
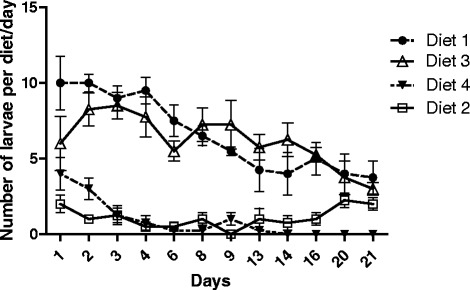
Number of larvae on four different diets over time. Diet 1 (rabbit feces, rabbit food, fish food, and soil); 2 (rabbit feces and soil); 3 (rabbit feces, soil and fish food); and 4 (soil and mud). *P* < 0.05

According to the results, the most attractive larval food was diet 1, followed by diet 3 (Fig. 2). Both diets contained fish food. Differences were significant among the four diets (*p* < 0.05), but there were no significant differences between diets 1 and 3 (*p* > 0.05) or 2 and 4 (*p* > 0.05). The presence of fish food appears to be an important factor in the attractiveness of a diet to the larval instars of *Ny. neivai.*

### Attractiveness and development of *Ny. neivai* larvae on two different diets

**Fig. 3:**
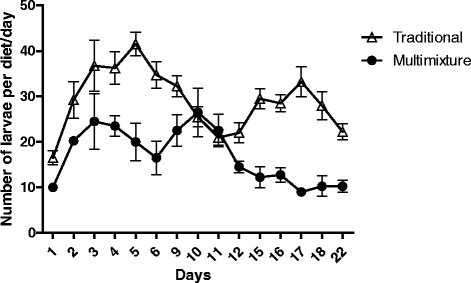
Number of larvae on two different diets over time. Traditional diet (rabbit feces, rabbit food, fish food, and soil) and multimixture diet (plants and grains). *P* < 0.05

The number of larvae on diet 1 (traditional) was higher than on the multimixture (*p* < 0.05) (Fig. 3).

The productivity of adults was much higher on diet 1 (164 adults; 41 %) than on the multimixture (19; 9.5 %) (*p* > 0.05).

### Development of immature stages on non-sterilized and sterilized diets

**Fig. 4:**
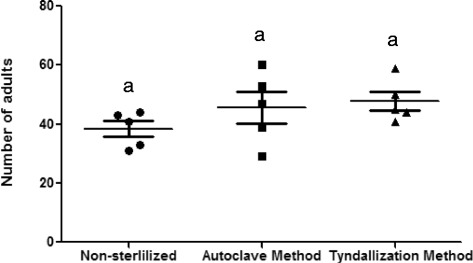
Emergency of adults on non-sterilized and sterilized diets. Means with same letters are not significantly different (P>0.05)

There were no significant differences among the treatments (*p* > 0.05). However, the means were higher with both of the sterilized methods, autoclave (46.7 insects) and tyndallization (47.8 insects), compared to only 38.4 insects in the non-autoclaved diet (Fig. 4).

### Evaluation of different substrates for rearing the colony

**Fig 5:**
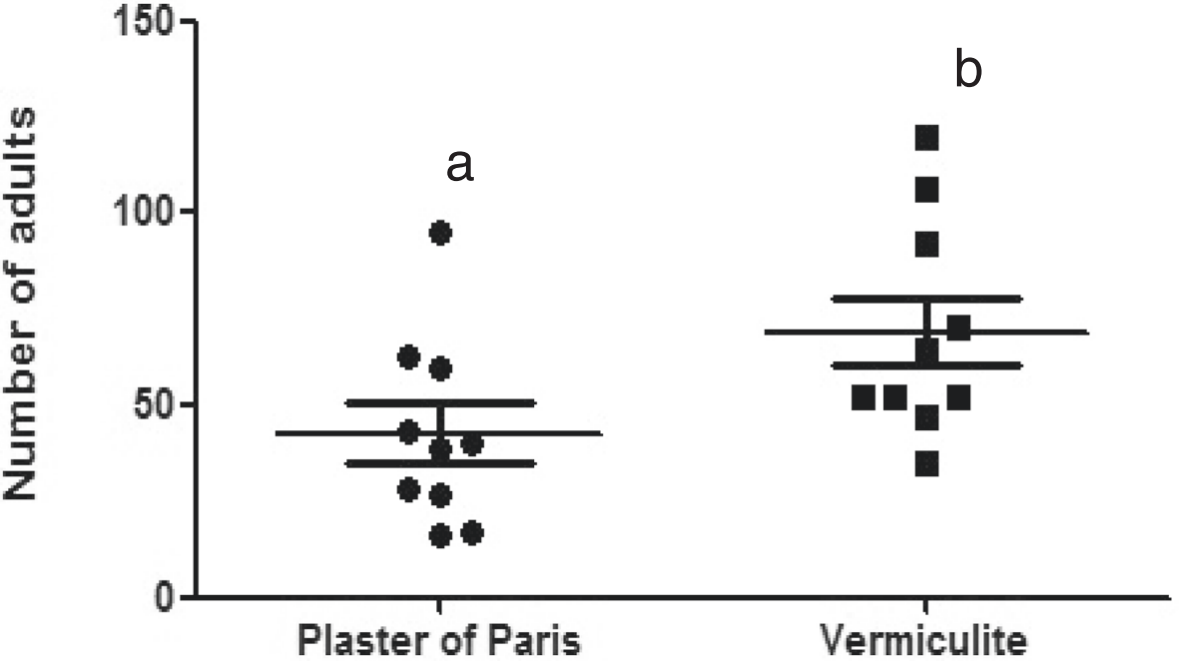
Emergency of adults in containers with two different substrates: plaster of Paris or vermiculite. Means with different letters are significantly different (P<0.05)

There was a significant difference between the plaster of Paris and the vermiculite (*p* < 0.05). The mean emergence of adults on the vermiculite was 690 insects, compared to 428 insects on the plaster of Paris (Fig. 5).

## Discussion

Rearing sand fly colonies in the laboratory provides the opportunity to improve the techniques for obtaining a large number of insects for basic or applied studies. Many aspects of the rearing process need to be considered for the success of a laboratory colony, such as carrying capacity, the right conditions for the immature stages, diet and substrate.

The concept of evaluating carrying capacity consists of determining the ideal number of individuals for a specific environmental condition [10, 11].

The maximum number of couples used in this work was 30, and with that number, the density of couples did not affect the mortality of females prior to oviposition. For *Ny. neivai*, our results showed that the carrying capacity was between five and ten couples for both sizes of containers (150 and 250 mL), where the highest number of adults were achieved.

Santamaría et al. studied the carrying capacity of *Pintomyia serrana* and verified that the density of flies negatively affected the survival of the females prior to oviposition [11]. However, those authors worked with 1 to 50 couples, which is more than the number of pairs used in our experiments. Their best productivity was with 22 females per container. Under those conditions, female mortality was low and the numbers of eggs and adults emerged were the highest. Apart from the different experimental conditions between the studies, it is probable that other specific characteristics could be involved.

A previous study with a colony of *Ny. neivai* showed a rate of adults emerged of 20.7 % [12]. Our results showed that a lower number of couples inside the rearing container produced a higher number of adults (Fig. 1). This could be explained by the intraspecific competition for food and space among the larvae. When the container is too crowded, the larvae climb the walls and end up dying of dehydration. The same behaviour was observed by Hertig & Johnson, who called this phenomenon a “suicidal mass migration” [13]. Experiments with *Culex sitiens* showed that when subjected to overcrowding, the larvae compete for space to feed, increasing their mortality [14].

Many authors have been studying diets for the immature stages of different sand fly species. A mixture of aged rabbit feces and rabbit food was used to rear *Lutzomyia cruciata*, *Dampfomyia anthophora*, *Psathyromyia shannoni,* and *Micropygomyia vexator* under laboratory conditions. This diet was successful in reducing the growth of fungi [15]. Some modifications of this diet have been used in many sand fly colonies: *Phlebotomus papatasi*, *Phlebotomus argentipes, Phlebotomus duboscqi*, *Lutzomyia longipalpis* [16, 17] and *Pi. serrana* [2].

Industrialized animal food (for rabbits, dogs, hamsters and aquarium fishes) was compared with aged food for larvae of *Nyssomyia intermedia* and *Lu. longipalpis*. Both species developed very well with the aged food, but unlike *Lu. longipalpis*, *Ny. intermedia* also presented good results with industrialized animal food [4]. It has been suggested that variability in sand fly species dietary preferences can be important in reducing competition under natural conditions [18].

We did not use aged food in this study. Our results showed that diets that were enriched with fish food were more attractive to sand fly larvae compared to ones without this component. This observation is in agreement with the previous experiments of Rangel et al. [19], and this should be investigated in further studies on the possible attractants for larvae. In our experiments, the survival of adults was higher with traditional food (rabbit feces, rabbit food, fish food and soil) than with other diets that were made up only of plants and different grains (multimixture diet). The multimixture also presented a huge growth of fungi, which cause damage to the lifecycle of the insect. The fungi that grew on the multimixture were extremely dense and had to be removed daily during the insect counting. There is a controversy surrounding the relevance of fungus in diets for sand flies. According to some authors, the fungi on the diet could be fatal to the early instar of the larvae by trapping them on fungi hyphae [13, 18, 20]. Otherwise, the fungi can be profitable for the larvae, complementing their nutrition [4].

Considering the disagreement of many authors about fungi growth on sand fly diets, we decided to compare non-sterilized and sterilized diets. Even though our results did not show a significant difference between sterilized and non-sterilized diets, the autoclave and tyndallization methods (sterilized diets) retarded fungal growth for at least three days, giving enough time for the first instar larvae to develop. Under these conditions, the first instar larvae can grow a little more and avoid being trapped by the hyphae.

Some sand fly species of the *Phlebotomus* genus prefer non-autoclaved food, but for other species, like *Lu. longipalpis*, the food can be autoclaved [2]. We observed that the larvae of *Ny. neivai* did not refuse, in any stage, the sterilized diet, which had the advantage of a decrease in fungi growth; thus, we strongly advise the sterilization method for fungal control in *Ny. neivai* colonies.

The oviposition experiments on different substrates showed that an irregular surface (vermiculite) presented a higher productivity of adults compared to a flat surface (plaster of Paris). The gravid females were able to oviposit among vermiculite grains. The vermiculite also provided a “refuge” from the fungi hyphae among the grains for the first larval instars. Shaking the containers with grains would break the hyphae. Studies on the physical factors showed that temperature and humidity are important factors in the oviposition behaviour of *Ny. neivai* [21, 22]. Rough and irregular substrates stimulate a thigmotropic response in gravid flies of *L. longipalpis* and *L. migonei* and also provide greater protection, higher humidity and possibly more food for newborn larvae than flat surfaces [23, 24].

## Conclusion

In conclusion, our data on *Ny. neivai* colonization showed a variable emergency rate of adults in different tests, depending on larval population density, diet and oviposition surface. The best adult productivities were achieved with fewer couples inside the rearing chambers (400–600 eggs depending on the size of the container); smaller rearing containers of 150 mL (due to less fungi growth); sterilized diet made of rabbit feces, rabbit food, soil and fish food; and vermiculite as the substrate for oviposition and development of larvae.
